# Supplementary material for: Involving men in cervical cancer prevention; a qualitative enquiry into male perspectives on screening and HPV vaccination in Mid-Western Uganda
Source: PLoS One. 2023 Jan 27;18(1):e0280052. doi: 10.1371/journal.pone.0280052 (PMC9882699; doi:10.1371/journal.pone.0280052)
Supplement: S1 File — (DOCX) [file pone.0280052.s001.docx]

**S1file. Focus group discussion guide**

*Introduction of moderators and FGD*

Female reproductive health

1. Do you talk about diseases that affect women; like heavy menstrual bleeding, vaginal discharge, STI’s or pregnancy-related diseases? With whom?

Cervical cancer

1. How can women get cervical cancer? What is the underlying process?
2. How does cervical cancer affect women in your community?

*(prompt: physically, psychologically, socially, fear for witchcraft)*

1. Is it possible to prevent cervical cancer? How can it be prevented?
   *(Ask where they got this information. If not mentioned ask what they have heard about vaccination.)*

***Explain cervical cancer screening and HPV vaccination.***

Screening and vaccination program participation

1. How do you think about the genital examination of your wife?
   Does it matter who performs it?
2. What are barriers for you and your wives for screening/treatment?
3. Who decides if women should go for screening?
   *(If not mentioned, ask about the role and consent of husbands/partners)*Who decides about HPV vaccination of your daughter?

*(If not mentioned, ask about the role of the teacher)*

1. Do you discuss HPV-vaccination with your daughter or niece?
   If no, who do you think she would discuss it with?

*Do you think HPV-vaccination can result into promiscuous behavior of young girls?*

*What are barriers for you/your wife/your daughter for HPV vaccination?*

Perception on screening results

1. How will you react if your wife tests positive during screening? What would you do?

Do you think the community will find out about the results?
How do you think the community would react?

*Can the community also have a supporting role? In what way?*

Messages cervical cancer prevention

1. What message would you give other men from the community about cervical cancer prevention?

How would you explain prevention to your neighbor? In what form would they want to receive health education? (*Radio/Information days/brochures)*

*At the end:*

*Put 2 jars in a quiet place close to the FGD location in which participants can drop a bean (or anything locally available) to count how many participants would accept screening and HPV vaccination for their relatives.*
