# Supplementary material for: Involving men in cervical cancer prevention; a qualitative enquiry into male perspectives on screening and HPV vaccination in Mid-Western Uganda
Source: PLoS One. 2023 Jan 27;18(1):e0280052. doi: 10.1371/journal.pone.0280052 (PMC9882699; doi:10.1371/journal.pone.0280052)
Supplement: Attachment — Submitted filename: Rebuttal letter PONE-D-22-00308.docx [file pone.0280052.s003.docx]

**Attn PLOS ONE Editors**

Re: Rebuttal letter manuscript PONE-D-22-00308

August 30, 2022

Dear Editors and Reviewers

We are pleased with your positive response to our manuscript “Involving men in cervical cancer prevention; a qualitative enquiry into male perspectives on screening and HPV vaccination in Mid-Western Uganda” and hereby submit our revised manuscript. We apologize for our delayed response due to maternity leave of the corresponding author. Please find below our response to your valuable suggestions.

| **S/N** | **Comment** (summarized) | **Response** |
| --- | --- | --- |
| Journal 1. | Please ensure that your manuscript meets PLOS ONE's style requirements, including those for file naming. | We have adjusted our manuscript to the PLOS ONE style recommendations, as mentioned on the website. |
| Journal 2. | Please include a complete copy of PLOS’ questionnaire on inclusivity in global research in your revised manuscript. | Kindly find the completed questionnaire as supplementary file. |
| Journal 3. | Our staff editors have determined that your manuscript is likely within the scope of our Cancer and Social Inequity Call for Papers. If you would like your manuscript to be considered for this collection, please let us know in your cover letter and we will ensure that your paper is treated as if you were responding to this call. | Thank you for your suggestion to consider our manuscript for this interesting and very relevant special issue. Unfortunately, the deadline for submission for this special issue was 8 March 2022. If there is still an opportunity for publication in a related special issue we would be willing to consider. |
| Journal 4. | Please provide an amended statement that declares *all* the funding or sources of support (whether external or internal to your organization) received during this study, as detailed online in our guide for authors at http://journals.plos.org/plosone/s/submit-now.  Please also include the statement “There was no additional external funding received for this study.” in your updated Funding Statement.  Please include your amended Funding Statement within your cover letter. We will change the online submission form on your behalf. | Please find our updated financial statement below.  This research was supported by funding of Dioraphte Foundation (<https://www.dioraphte.nl/>), a non-commercial foundation from The Netherlands, granted to MF and JB via the Female Cancer Foundation (<https://www.femalecancerfoundation.org/>). The funders had no role in study design, data collection and analysis, decision to publish, or preparation of the manuscript. There was no additional external funding received for this study. |
| Journal 5. | Please review your reference list to ensure that it is complete and correct. | We reviewed our reference list and updated the references and their lay-out with track changes in our revised manuscript according to PLOS ONE guidelines.  We did not identify incorrect or retracted references. |
| Reviewer 1 | I would suggest condensing Figure 1 and its current format is more of a distraction than beneficial to the background of the paper. | Thank you for your suggestion to make the table more informative. We have removed the last column of the table (number of participants with a relative with cervical cancer) and the median in the column with number of daughters per relatives. We also combined two categories of educational level (secondary and higher education) in a percentage as this is most relevant. |
| Reviewer 1 | In the results section the quotes chosen for specific themes did not always seem intuitive and sometimes even detracted from what was being emphasized, perhaps the theme itself was a bit of a stretch or a different quotation could be used to ensure a more coherent message | Thank you for your comment about the quotations we selected. We have reviewed all quotations with the team of authors following your review and discussed about their relevance for each theme, it is one of the challenges in qualitative research. We decided to delete some quotes which could distract the reader and which was already sufficiently reflected in the text. For some themes the topics mentioned were indeed surprising. On the other hand, we used the themes in our FGD guide to structure the discussion but followed the topics and discussion the men raised during the FGD. Therefore the topics and quotes might sometimes not feel entirely coherent. Furthermore, the English grammar of some quotes is not entirely correct but we decided to keep the quotes as close to the original version as possible. We feel that the current quotes do reflect the perspective of men as expressed during the different FGDs. |
| Reviewer 1 | Finally there has been other work in Uganda looking at men's attitudes to screening including the ASPIRE group (Understanding Men’s Perceptions of Human Papillomavirus and Cervical Cancer Screening in Kampala, Uganda, Moses et al 2018) which would be a good reference to add. | Thank you for this literature suggestion, this manuscript is included as reference number 25. |
| Reviewer 2 | As documented in the limitation - "We are aware that socially desired answers cannot be ruled out and that they can mask taboos. The large acceptance of screening after the FGD might not be reflected by screening of their wives in practice" - Was there any suggestion from the FGDs that could mitigate against this threat? This would provide further opportunities that can be exploited to improve practice of screening. I'm concerned that the method of participant selection may have contributed to the suspicion of "socially desired answers" and this may not allow for further interrogation on the possible barriers to increasing uptake. | Thank you for your remark and suggestions for future research in this field. During the FGDs we noticed active engagement of all participants with good interaction between the participants themselves and the participants and the moderators. After the FGDs men indicated that they felt relieved they could talk about the subject and that we were seriously interested in their perspective. From our experience in the screening project in Kagadi we found that many men would want their wives to be screened and stay healthy, but lack of knowledge about cervical cancer and the impact of screening, and poor access to screening are important barriers for their support. We purposely selected different age groups because in this context younger men will not feel comfortable to speak freely out of respect for elders. In addition, we had a separate FGD with influential men in the community.  In future research we would consider approaching husbands of women that did not participate in screening, where this has been actively offered to all women in the community, for in-depth interviews. Another alternative could be to ask women coming for screening if they felt supported by their men, and approach their men for in-depth interviews (from both women who felt supported and who felt not supported), however, this will be difficult in practice to obtain consent and to get in contact with them.  In order to gain more insight, we hope our research shows that men like to be involved and therefore we advise to include men from the beginning in cervical cancer awareness and programming of prevention, and involve them actively in community engagement. This will also help to better understand their perspective and possible barriers and opportunities. |

We trust we have replied to your queries and want to thank you for your suggestions which improved our initial manuscript to its current version. Please do not hesitate to contact us in case of any further questions. We look forward to hearing from you.

Yours sincerely,

Marlieke de Fouw

[marlieke@femalecancerfoundation.org](mailto:marlieke@femalecancerfoundation.org)
