# Supplementary material for: Involving men in cervical cancer prevention; a qualitative enquiry into male perspectives on screening and HPV vaccination in Mid-Western Uganda
Source: PLoS One. 2023 Jan 27;18(1):e0280052. doi: 10.1371/journal.pone.0280052 (PMC9882699; doi:10.1371/journal.pone.0280052)
Supplement: Attachment — Submitted filename: Rebuttal letter PONE-D-22-00308R1.docx [file pone.0280052.s004.docx]

**Attn PLOS ONE Editors**

Re: Rebuttal letter manuscript PONE-D-22-00308R1

November 23, 2022

Dear Editors and Reviewers

We thank you for your review and careful consideration of our revised manuscript “Involving men in cervical cancer prevention; a qualitative enquiry into male perspectives on screening and HPV vaccination in Mid-Western Uganda”. Please find below our response to your suggestions, hereby we submit our revised manuscript.

| **S/N** | **Comment** (summarized) | **Response** |
| --- | --- | --- |
| Journal requirement | Please review your reference list to ensure that it is complete and correct. | We reviewed our reference list and updated the references and their lay-out with track changes in our revised manuscript according to the PLOS ONE guidelines. We did not identify incorrect or retracted references.  In addition to our previous rebuttal letter, we would like to clarify our revisions:   1. The first reference of Gakidou et al, was replaced by Bruni et al, because this is the most recent publication about the coverage of cervical cancer screening in LMICs and this paper was not yet available at the time of our initial submission. 2. For reference 8 we included the updated link, to access the National Strategic Plan of Uganda. 3. The other revisions are the adjustment of the presentation of the journal titles to the uniform Vancouver system. |
| Additional Editor Comments | While the authors addressed the original reviewer comments in the response document, not all translated to revisions of the manuscript itself. Specifically, I would like to see the manuscript edited to reflect the question/discussion on the final reviewer item, especially since the information provided by the authors with regards to age-based selection and additional FGD's with influential participants isn't outlined/consistent with methods. | Thank you for your suggestion to edit our manuscript to bring these considerations to the attention or the reader, and not only the reviewers. We have adjusted the relevant sections in methods, results, and discussion in track changes. |

Please do not hesitate to contact us in case of any further questions. We look forward to hearing from you.

Yours sincerely,

Marlieke de Fouw

[marlieke@femalecancerfoundation.org](mailto:marlieke@femalecancerfoundation.org)
